# FTO plays a crucial role in gastrointestinal cancer and may be a target for immunotherapy: an updated review

**DOI:** 10.3389/fonc.2023.1241357

**Published:** 2023-10-12

**Authors:** Xiangqing Ren, Xiaolong Tang, Tian Huang, Zenan Hu, Yuping Wang, Yongning Zhou

**Affiliations:** ^1^ The First Clinical Medical College, Lanzhou University, Lanzhou, China; ^2^ Department of Gastroenterology, The First Hospital of Lanzhou University, Lanzhou, China; ^3^ Gansu Province Clinical Research Center for Digestive Diseases, The First Hospital of Lanzhou University, Lanzhou, China

**Keywords:** FTO, m6A, gastrointestinal cancer, immunotherapy, tumor microenvironment

## Abstract

Gastrointestinal cancer is a common malignancy with high mortality and poor prognosis. Therefore, developing novel effective markers and therapeutic targets for gastrointestinal cancer is currently a challenging and popular topic in oncology research. Accumulating studies have reported that N6-methyladenosine is the most abundant epigenetic modification in eukaryotes. N6-methyladenosine plays an essential role in regulating RNA expression and metabolism, including splicing, translation, stability, decay, and transport. FTO, the earliest demethylase discovered to maintain the balance of N6-adenosine methylation, is abnormally expressed in many tumors. In this review, we discuss the molecular structure and substrate selectivity of FTO. we focus on the role of FTO in gastrointestinal tumor proliferation, migration, invasion, apoptosis, autophagy, immune microenvironment, and its molecular mechanisms. We also discuss its potential in the treatment of gastrointestinal cancers.

## Introduction

Gastrointestinal cancer (GIC), which includes esophageal cancer (EC), gastric cancer (GC), colorectal cancer (CRC), pancreatic cancer (PC), liver cancer (LC), and biliary tract cancer (BTC) (1), is a significant cause of cancer-related mortality and remains a leading challenge in cancer treatment (2). Despite the integrated treatment of chemoradiotherapy(CRT) and modern surgical techniques, the overall 5-year survival rate of patients with advanced GIC is under 15%, owing to rapid disease progression, metastasis, and CRT resistance (3). However, immunotherapy based on checkpoint inhibitors has shown an excellent tumor-suppressive effects in clinical studies, offering a bright prospects in cancer treatment (4).

Anti-programmed cell death protein 1/programmed cell death ligand 1 (anti-PD-1/PD-L1) and anti-cytotoxic T-lymphocyte-associated protein 4 (anti-CTLA-4) are the most commonly used tumor immunotherapy approaches (4). Low cytotoxicity, long-term tumor regression, and prevention of recurrence make this novel treatment strategy a promising candidate for cancer treatment (4, 5). Immune checkpoint inhibitors (ICIs) have transformed the treatment landscape for various cancers, including GIC. However, only 20-25% of patients respond to ICIs (5), indicating the urgent need to identify effective biomarkers to screen patients who may benefit from ICIs.

N6-methyladenosine (m6A), as the most abundant post-transcriptional modification of RNA, plays an important role in various malignant tumors (6, 7). FTO, a member of the a-ketoglutarate-dependent dioxygenase family, regulates cellular energy metabolism. Multiple malignant tumors are associated with FTO dysregulation, which is involved in various pathological processes (8, 9). In a landmark study, overexpression of FTO promoted leukemia progression and inhibited all-trans-retinoic acid-induced acute myeloid leukemia (AML) differentiation by reducing m6A levels in ASB2 and RARA mRNA (10). Subsequently, several researchers demonstrated that FTO overexpression induced GIC in humans. FTO stabilized MYC mRNA by reducing m6A methylation in GC cells, thereby promoting GC development (11). FTO regulated mRNA stability through demethylation of G6PD/PARP1, promoting CRC progression and chemotherapy resistance (12). Many clinical studies have reported that targeting FTO can significantly improve the prognosis of patients with GC, CRC, and other GIC (13–17). These results suggested that FTO inhibition may be an effective treatment strategy for GIC.

Specifically, the role of FTO in the tumor immune microenvironment has attracted interest. Su et al (18). have shown that highly expressed FTO can inhibit the immune activity of T lymphocytes by increasing the expression of the immunosuppressive checkpoint molecule/gene “LILRB4”, thereby promoting the progression of leukemia stem cells. This review focuses on how FTO promotes the progression of gastrointestinal malignancy and regulates the expression of immune checkpoints and immune cells activity.

## Introduction to FTO

FTO is first identified in the Genome-wide Association Study (GWAS) on obesity and type 2 diabetes (8, 19). It encodes the FTO protein belonging to the Fe^2+^ and 2-oxoglutarate (2OG)-dependent AlkB dioxygenase family (20–22). Human FTO is 410.50 KB long, including eight introns and nine exons, and is located on chromosome 16Q12.2 (23). Regarding the protein molecular structure, FTO consists of two domains, a catalytic N-terminal domain (NTD, residue 32-326) and a C-terminal domain (CTD, residue 327-498) (8, 24). NTD consists of a double-stranded β-helix fold called the jelly-roll motif (residues 201-322). The three conserved residues in this motif serve as catalytic domains containing metal ion. The hydrogen bonds formed by N205, Y295, R316, S318, and R322 stabilize N-oxalylglycine (NOG), a bidentate ligand (8, 25). CTD is primarily composed of α-helix. Notably, one three-helix bundle interacts extensively and closely with the NTD, and mutations in specific amino acids involved in these interactions (F114D or C392D) significantly reduce the physiological activity of FTO, suggesting that the CTD plays a vital role in stabilizing the conformation and catalytic function of the NTD (8, 26). FTO uses cofactors 2OG and Fe^2+^ to catalyze m6A removal and progressively generates N6-hydroxymethyl adenosine and N6-formyl adenosine (27). Han et al (24). revealed the substrate specificity of the FTO protein by analyzing its crystal structure and found that the single nucleotide 3-meT/3-meU contained all the basic structural determinants for FTO to recognize its substrate. Zhang et al. (25) further revealed the activity preference of FTO for cap m6A over internal m6A in ssRNA and m1A in tRNA or loop-structured RNA over m1A in linear ssRNA by studying RNA sequences and tertiary structures. FTO affected snRNA m6A and m6Am levels and mediated tRNA m1A demethylation in various cells (28). However, due to the large heterogeneity of different species, as well as tissue and cell specificity, the function of m6A still needs further investigation (29–31).

At the cellular level, FTO is located in both the nucleus and cytoplasm, possibly shuttling between the two cellular compartments via a mechanism mediated by one of the exportin 2(XPO2) proteins (32, 33). Previous studies have shown that FTO binds to unmethylated double-stranded DNA and performs essential physiological functions (24). Wei et al. (34) found that FTO mediated m6A demethylation of long dispersing element-1 (LINE1) RNA in mouse embryonic stem cells, regulating the abundance of LINE1 RNA and local chromatin state, and thereby modulated the transcription of LINE1-containing genes. Further research has shown that FTO had a high affinity for m6A in mRNA and showed highly efficient and reversible demethylation activity (35). FTO plays a crucial role in the post-transcriptional regulation of RNA in splicing, nuclear production, degradation, and translation because of its extensive presence and dynamic coding (23, 36–38).

## FTO and GIC

Recent studies have shown that FTO is closely associated with proliferation, metastasis, invasion, apoptosis, chemotherapy resistance, and glucolipid metabolism of gastrointestinal tumor cells. In this study, we summarize the latest findings on FTO in GIC (**Table 1**).

**Table 1:** The key role of FTO in GIC.

cancer	role	targets	Mechanism	function	refs
ESCA	Oncogene	SIM2	FTO reduced the stability of SIM2 mRNA by reducing the m6A methylation level of SIM2.	proliferation apoptosis	(39)
	Oncogene	MMP13	FTO positively regulated the mRNA and protein expression levels of MMP13.	proliferation migration	(40)
	Oncogene	LINC00022	FTO increased LINC00022 mRNA stability.	proliferation	(41)
GC	Oncogene	ITGB1 FAK	FTO promoted ITGB1 mRNA expression, and enhanced the phosphorylation of FAK	migration invasion	(14)
	Oncogene	Caveolin-1	FTO enhanced the degradation of Caveolin-1 mRNA	proliferation, migration, invasion	(42)
	Oncogene	mTORC1 DDIT3	FTO negatively regulated DDIT3 in an m6A-dependent manner	autophage apoptosis	(43)
	Oncogene	Wnt And PI3K/Akt signaling pathways	FTO activated Wnt and PI3K/Akt signaling pathways	proliferation migration invasion	(44)
	Oncogene	MYC	FTO stabilized MYC mRNA by removing the m6A modification	proliferation migration invasion	(11)
CRC	Oncogene	G6PD PARP1	FTO regulated the expression level of G6PD/PARP1 through m6A	proliferation	(12)
	Oncogene	MZF1	FTO promoted MZF1 expression in an m6A-dependent manner	proliferation migration apoptosis	(45)
	Oncogene	ATF4	FTO-induced ATF4 promoted pro-survival autophagy	pro-survival autophagy	(46)
PLC	Oncogene	PKM2	FTO led to the demethylation of PKM2 mRNA, promoting its mRNA production and accelerating its translation process	proliferation apoptosis	(47)
	Oncogene	NANOG SOX2 KLF4	FTO significantly promoted the expression of NANOG, SOX2 and KLF4 in HCC cells by mRNA demethylation	HCC cells stemness	(48)
	Oncogene	ERa	Reduced FTO can increase the m6A level of estrogen receptor alpha (ERa) mRNA, thereby reducing the protein translation of ERa	proliferation	(49)
PAAD	Oncogene	c-MYC	FTO improved the stability of c-MYC expression	proliferation	(50)
	Oncogene	TFPI-2	FTO inhibited the stability of TFPI-2 mRNA through the m6A reader YTHDF1	proliferation, migration, invasion	(51)

## FTO and EC

EC is among the top ten most common malignancies worldwide, ranking as the sixth most common cancer according to the 2020 tumor-related mortality rates (52, 53). Over 600,000 individuals worldwide are diagnosed with EC annually. The 5-year survival rate of EC is less than 20% (54). Esophageal squamous cell carcinoma (ESCC) and esophageal adenocarcinoma (EAC) are two common histological subtypes (55). FTO is overexpressed at the cellular and tissue levels of ESCC, and is significantly associated with poor clinical prognosis (39–41, 56).

**Figure 1:**
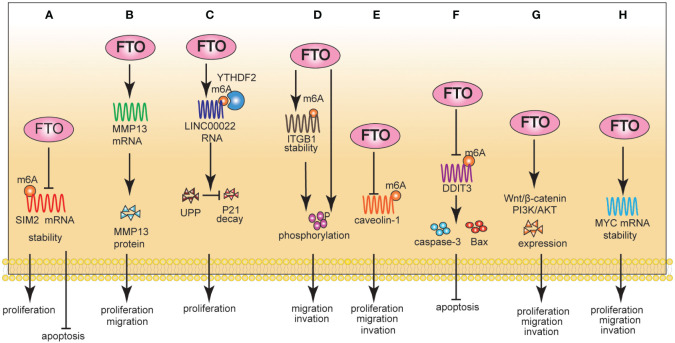
The Role of FTO in EC **(A-C)** and GC **(D-H)**. A: Mechanically, FTO reduced the stability of SIM2 mRNA by reducing the m6A methylation level of SIM2; Phenotypically, FTO promoted proliferation and inhibited apoptosis. B: Mechanically, FTO positively regulated mRNA and protein expression levels of MMP13; Phenotypically, FTO promoted proliferation and migration. **(C)**: Mechanically, FTO increased LINC00022 mRNA stability; Phenotypically, FTO promoted proliferation. **(D)**: Mechanically, FTO promoted ITGB1 mRNA expression, and enhanced the phosphorylation of FAK. Phenotypically, FTO promoted proliferation and invasion. **(E)**: Mechanically, FTO enhanced the degradation of Caveolin-1 mRNA. Phenotypically, FTO promoted proliferation, migration, and invasion. **(F)**: Mechanically, FTO negatively regulated DDIT3 in an m6A-dependent manner. Phenotypically, FTO inhibited apoptosis. **(G)**: Mechanically, FTO activated Wnt and PI3K/Akt signaling pathways. Phenotypically, FTO promoted proliferation, migration, and invasion. **(H)**: Mechanically, FTO stabilized MYC mRNA by removing the m6A modification. Phenotypically, FTO promoted proliferation, migration, and invasion.

Based on the detailed molecular mechanism, FTO can reduce the stability of SIM2 mRNA by reducing its m6A abundance, promoting the proliferation of ESCC cells and inhibiting cell apoptosis (39). In contrast, FTO positively regulated the mRNA and protein expression of MMP13, thus promoting the proliferation and migration of ESCC cells (40). However, it is still unclear whether FTO regulated the expression level of MMP13 in an m6A-dependent manner; therefore, the interaction between the two molecules needs further research. Methylation of lncRNAs by FTO is rare. Recent studies have shown that FTO mediated m6A demethylation of LINC00022 to increase its RNA stability, thereby promoting its expression in a YTHDF2-dependent manner. Furthermore, LINC00022 promoted the decay of the P21 protein through the ubiquitin-proteasome pathway (UPP) (41) (**Figures 1A-C**).

## FTO and GC

Multiple clinical and bioinformatics studies have shown that FTO is significantly overexpressed at the cellular and tissue levels of GC and acts as an oncogenic gene to promote the progression of GC (13–16). The Cox proportional risk model showed that FTO expression, histological grade, TNM stage, invasion depth, and lymph node metastasis significantly correlated with the overall survival (OS) rate of patients with GC. Furthermore, FTO expression and TNM stage were independent prognostic indicators of OS in patients with GC (13). Wang et al. (14) found that FTO promoted ITGB1 mRNA expression by enhancing its stability in an m6A-dependent manner. Notably, FTO promoted the malignant progression of GC cells through the ITGB1-FAK pathway.

Furthermore, FTO enhanced the degradation of Caveolin-1 mRNA by reducing its m6A level, which regulated mitochondrial fission/fusion and metabolism. Finally, FTO promoted GC cell proliferation, migration, and invasion (42). WNT/β-catenin and PI3K/AKT/mTOR signaling pathways are critical regulators of essential biological functions in malignant tumors, including cell proliferation, metabolism, angiogenesis, and epithelial-mesenchymal transformation (EMT) (57–59). FTO promoted the proliferation, migration, and invasion of GC cells, possibly by activating WNT/β-catenin and PI3K/AKT/mTOR signaling pathways (44). However, this study only analyzed the correlation between FTO and key molecule protein expression in the signaling pathway. Therefore, the mechanism underlying FTO-mediated regulation of WNT/β-catenin and PI3K/AKT/mTOR signaling still needs further investigation. FTO stabilized MYC mRNA by removing m6A modifications, ultimately promoting the proliferation, migration, and invasion of GC cells. Notably, this study verified the upstream regulatory mechanism of FTO and found that HDAC3 promoted the expression of FTO and MYC by degrading FOXA2 (11) (**Figures 1D-H**).

FTO not only plays an important role in the development of GC, Feng et al. (43) found that FTO-mediated activation of mTORC1 and DDIT3 up-regulation was involved in the improved chemosensitivity of GC induced by omeprazole.

## FTO and CRC

**Figure 2:**
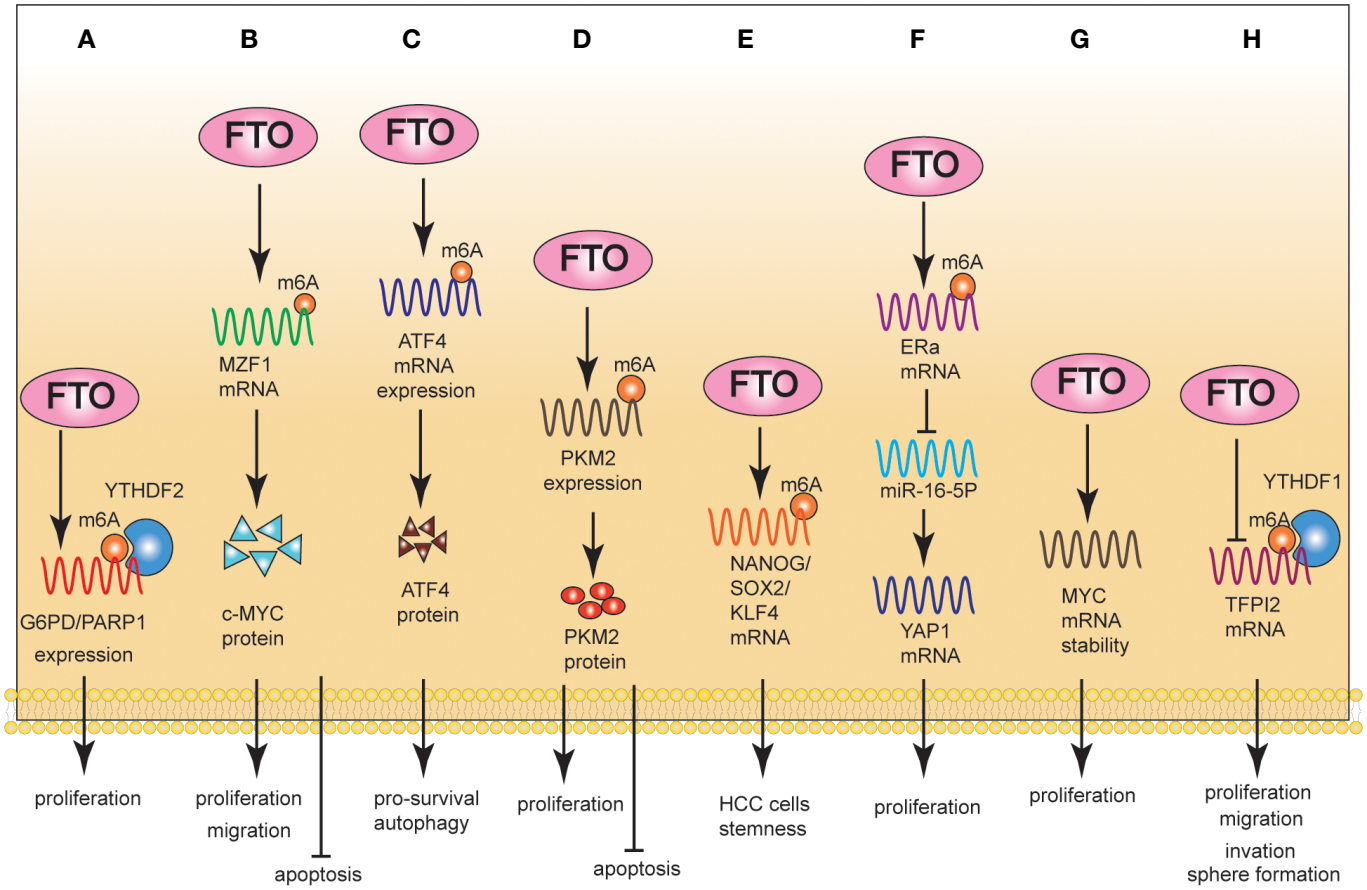
The Role of FTO in CRC **(A-C)**, PLC **(D-F)**, and PAAD **(G, H)**. **(A)**: Mechanically, FTO regulated the expression level of G6PD/PARP1 through m6A; Phenotypically, FTO promoted proliferation. **(B)**: Mechanically, FTO promoted MZF1 expression in an m6A-dependent manner; Phenotypically, FTO promoted proliferation/migration and inhibited apoptosis. **(C)**: Mechanically, FTO-induced ATF4 promoted pro-survival autophagy. **(D)**: Mechanically, FTO led to the demethylation of PKM2 mRNA, promoting its mRNA production and accelerating its translation process. Phenotypically, FTO promotes proliferation and inhibits apoptosis. **(E)**: Mechanically, FTO significantly promoted the expression of NANOG, SOX2 and KLF4 in HCC cells by mRNA demethylation. Phenotypically, FTO promoted HCC cell stemness. **(F)**: Mechanically, reduced FTO can increase the m6A level of estrogen receptor alpha (ERa) mRNA, thereby reducing the protein translation of ERa. Phenotypically, FTO promoted proliferation. **(G)**: Mechanically, FTO improved the stability of c-MYC expression. Phenotypically, FTO promoted proliferation. **(H)**: Mechanically, FTO inhibited the stability of TFPI-2 mRNA through the m6A reader YTHDF1. Phenotypically, FTO promoted proliferation, migration, and invasion.

One study showed that FTO mediated intracellular ROS balance by regulating the expression of G6PD and maintained genomic instability by regulating the expression of PARP1. Notably, FTO regulated the expression of G6PD/PARP1 through m6A manner. Targeting FTO can significantly inhibit cancer cell growth and improve sensitivity to chemotherapy (12). FTO enhanced the expression of MYC by removing m6A modifications, thereby inducing proliferation, migration, and inhibiting the apoptosis of CRC cells (45, 60). Glutaminolysis inhibition upregulated FTO to reduce m6A modification of activating transcription factor 4 (ATF4) mRNA. FTO-induced ATF4 expression promoted pro-survival autophagy in CRC cells (46) (**Figures 2A-C**).

## FTO and LC

FTO is upregulated in patients with hepatocellular carcinoma (HCC) and promotes HCC cells proliferation and migration. From a clinical perspective, FTO was an independent prognostic factor for HCC (17). Mechanistically, FTO led to the demethylation of PKM2 mRNA, promoting its mRNA production and accelerating its translation. FTO inhibited cell apoptosis and promoted proliferation through PKM2 demethylation (47). Notably, FTO enhanced HCC cell stemness in an m6A-dependent manner. The specific molecular mechanism was that a high level of AMD1 can increase the level of SPD in HCC cells, thus modifying the scaffold protein IQGAP1 and enhancing the interaction between IQGAP1 and FTO. This interaction can enhance the phosphorylation of FTO and reduce its ubiquitination, thus increasing FTO expression. Furthermore, FTO significantly promoted the expression of NANOG, SOX2, and KLF4 in HCC cells via mRNA demethylation (48). Gao et al. (49) found that FTO promoted cholangiocarcinoma (CCA) proliferation through the ERa/miR-16-5P/YAP1 signaling pathway (**Figures 2D-F**).

## FTO and PC

FTO is overexpressed in pancreatic adenocarcinoma (PAAD) cells and is critical for cancer progression (61). Regarding its underlying mechanism, FTO improved the stability of c-MYC expression, thereby promoting cell proliferation (50). Furthermore, FTO inhibited the expression of TFPI-2 mRNA through the m6A reader YTHDF1, leading to the down-regulation of TFPI-2 expression and ultimately promoting the proliferation, colony formation, sphere formation, migration, and invasion of PAAD cells, as well as tumor growth *in vivo* (51) (**Figures 2G, H**).

Single nucleotide polymorphisms (SNPs) are biomarkers of susceptibility to malignant tumors (62–64). The FTO rs9939609 polymorphism was associated with lung, kidney, and breast cancer (65, 66). Recent studies on FTO gene polymorphisms and PC have shown that the SNP of FTO, especially the FTO rs9939609 polymorphism, was significantly correlated with the occurrence of PC, suggesting that rs9939609 may be a potential biomarker for the early diagnosis of PC or a gene therapy target (67–70).

## Potential immunomodulatory effects of FTO

Currently, immunotherapy is at the forefront of cancer treatment. However, patients with GIC usually do not benefit as much as patients with other solid malignancies such as lung cancer and melanoma (3, 71). Advances in ICIs, especially anti-PD-1/PD-L1, and anti-CTLA-4, have enabled revolutionary progress in the treatment of malignant tumors (72). Recently, FTO has been confirmed to be closely related to the expression of multiple immune checkpoints in malignant tumors, making it a novel target with great potential.

## FTO modulates the immune microenvironment in CRC

Nobuhiro et al. (73) reported a positive correlation between the high expression of FTO and PD-L1 in CRC cells. In order to elucidate the underlying mechanism by which FTO regulated PD-L1 expression, FTO was knocked down in the presence of IFN-γ, the main stimulator of PD-L1 expression. The absence of FTO decreased PD-L1 expression in an IFN-γ independent manner. An RNA immunoprecipitation assay showed that m6A modified PD-L1 mRNA in CRC cells and the FTO bound to PD-L1 mRNA. In summary, m6A modification regulated the expression of PD-L1 in CRC cells, providing a novel approach for regulating PD-L1 expression via FTO modification.

Microsatellites are repeated DNA motifs widely distributed in the genome (74). If two or more replicates are altered, the tumor is defined as high-microsatellite instability (MSI-H); if only one mutation sequence is found, the tumor is considered to have low-microsatellite instability (MSI-L). A microsatellite located in a protein-coding region is called a coding microsatellite (cMS). Therefore, cMS mutations, also known as those encoding MSI (cMSI), are considered key events in the development of MSI cancer (75, 76).

**Figure 3:**
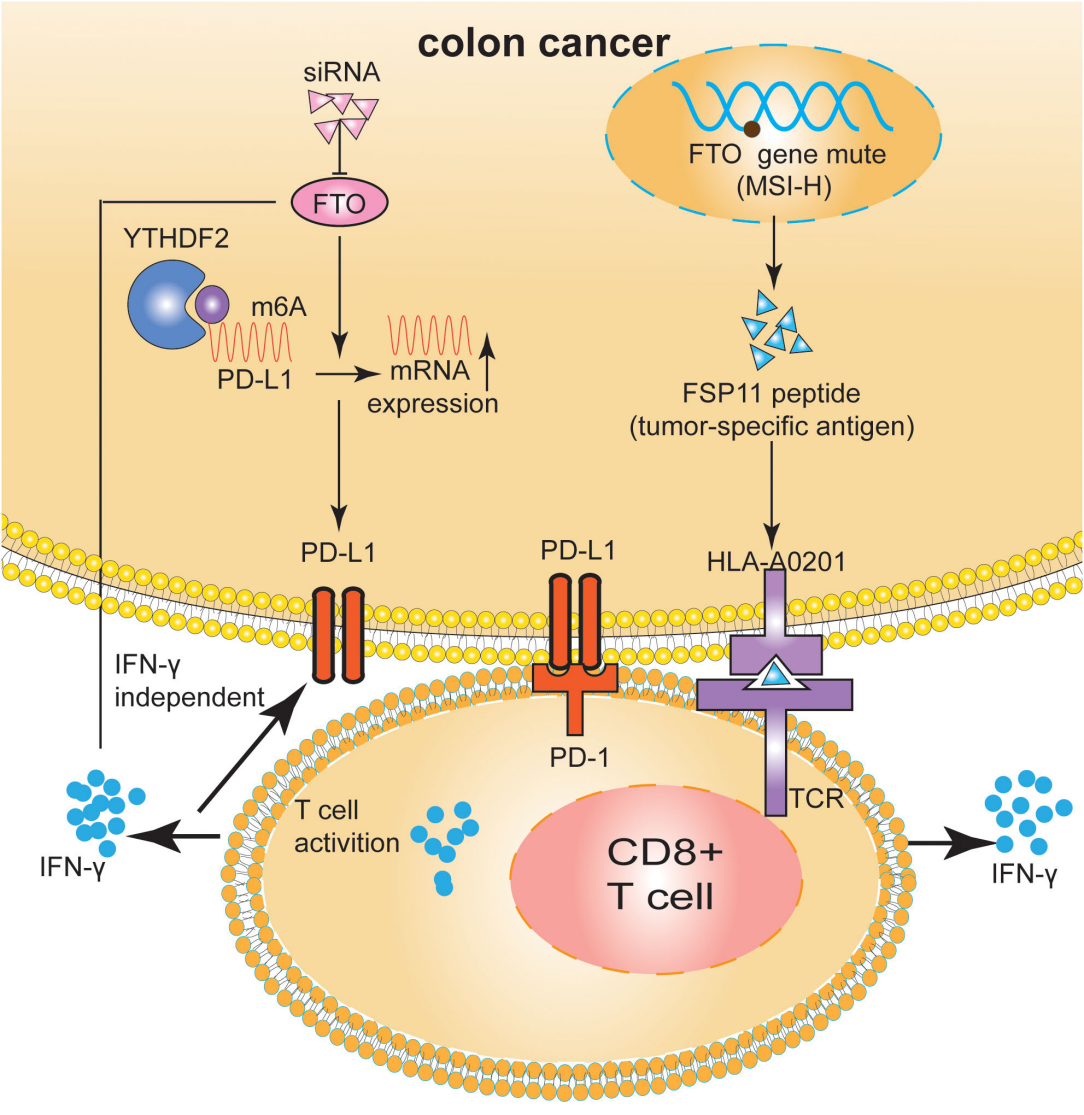
FTO regulated the tumor immune microenvironment in m6A-dependent manner and gene mutation in CRC. FTO increased PD-L1 mRNA expression levels in an IFN-γ independent manner. The HLA-A0201-restricted peptide FSP11 was derived from a (−1) mutation of U79260(FTO) gene. The peptide FSP11 sensitized peripheral CD8+ T cells for specific release of IFN-γ.

The mutation rate of U79260 (FTO) mRNA in colon cancer was very high, indicating high specificity for CRC cells. Frameshift peptide 11 (FSP11), a HLA-A0201-restricted peptide, was rooted in a (−1) mutation of THE T (15) channel in the U79260 gene. The peptide FSP11 sensitized the CD8^+^ T cells of peripheral blood for a specific release of IFN-*γ and* coordinated recognition in the context of HLA-A0201. Furthermore, the obtained T cell cultures selectively attacked cancer cells expressing potential mutations along with HLA-A0201 (77). MSI-H-induced FSP was a promising tumor-specific antigen that may be suitable for clinical applications in future tumor therapies (78). In addition to immunotherapy interventions for patients with MSI-H tumors, this opens up significant possibilities for preventive vaccination even for carriers of hereditary non-polyposis colorectal cancer (HNPCC) mutations (77–79) (**Figure 3**).

## FTO modulates the immune microenvironment in oral squamous cell carcinoma

**Figure 4:**
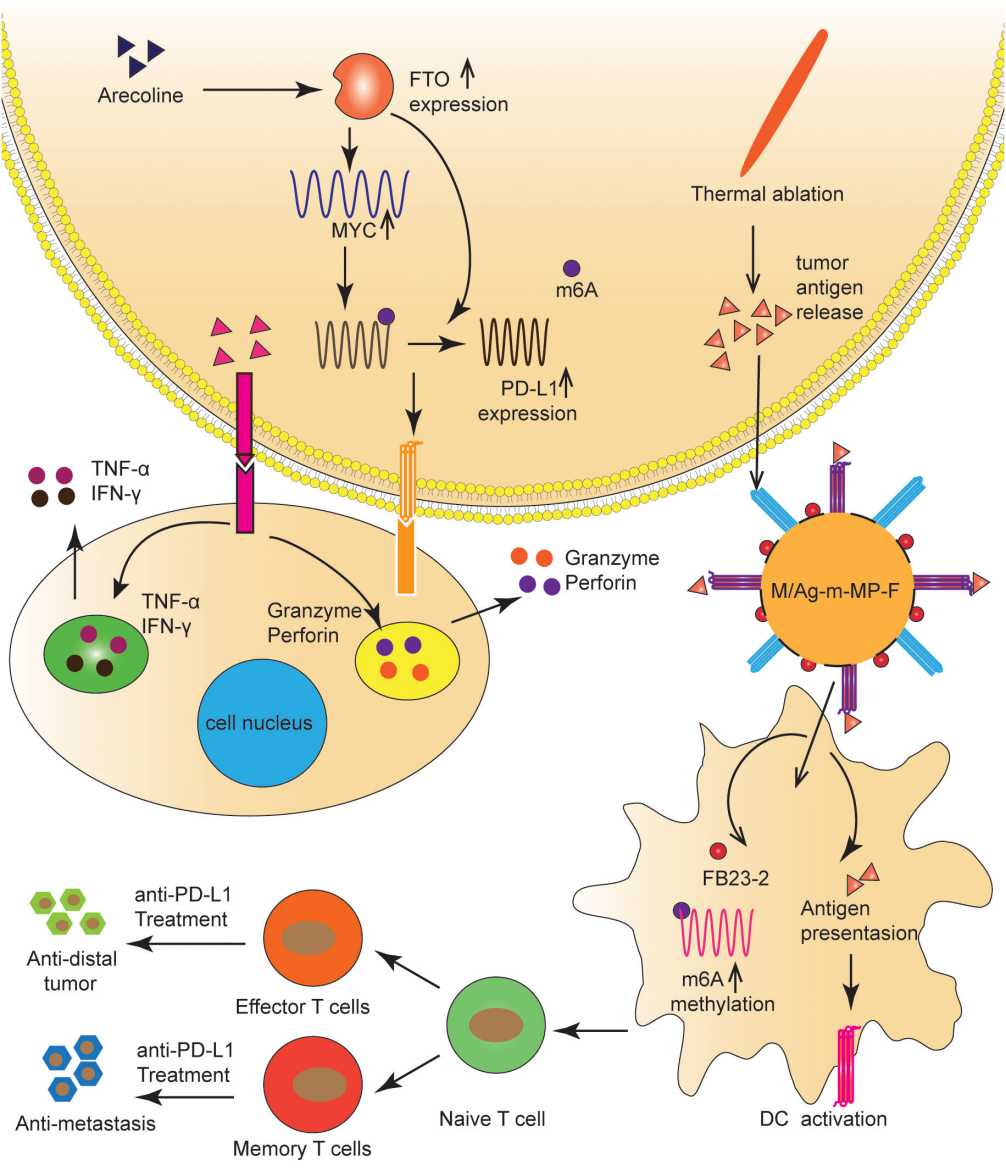
FTO regulated the tumor immune microenvironment in OC and HCC. FTO improved the stability of PD-L1 mRNA in an m6A-dependent manner, and MYC promoted PD-L1 transcription. TAAs and FB23-2 co-delivered to TIDCs were expected to help ICBs inhibit HCC progression by increasing m6A methylation, enhancing T-cell infiltration, and generating immune memory.

Arecoline is a primary carcinogen of oral squamous cell carcinoma (80, 81); however, its carcinogenic mechanism remains unclear (80). Li et al. (82) found that arecoline-induced FTO and MYC expression promoted the up-regulation of PD-L1 in oral squamous cell carcinoma cells. Specifically, FTO improved the stability of PD-L1 mRNA in an m6A-dependent manner, and MYC promoted PD-L1 transcription. PD-L1 upregulation enhanced cell proliferation, migration, and resistance to T-cell death. Therefore, arecoline plays a pro-cancer role by regulating FTO/MYC/PD-L1 signaling, which may be a strategy for immunotherapy (**Figure 4**). The mechanism by which FTO regulates PD-L1 needs further study.

## FTO modulates the immune microenvironment in LC

ICIs and thermal ablation (TA) have been extensively used to treat HCC (83). TA is known to release tumor-associated antigens (TAAs), which activate the anti-tumor immune response and initiate the infiltration of T cells (84). However, significant obstacles to incorporating ICIs with TA include the insufficient internalization of tumor antigens and the immaturity of tumor-infiltrating dendritic cells (TIDCs), which result in a poor immune response to tumor progression (83, 85). To address this problem, Xiao et al. (85) synthesized a novel nanomaterial in which the FTO inhibitor (FB23-2) was wrapped in the pores of mesoporous polydopamine (MPDA) nanoparticles, maleimide was used as an antigen catcher, and mannose as the active TIDCs-targeting ligand was fixed to the MPDA surface via a polyethylene glycol (PEG) ligand. Nanomaterials injected into the tumor captured TAAs released during TA and were then ingested by TIDCs via a mannose-mediated targeting effect. FB23-2 and TAAs co-delivered to TIDCs were expected to help ICBs inhibit tumor progression by increasing m6A methylation, enhancing T-cell infiltration, and generating immune memory (**Figure 4**).

## FTO modulates macrophage activation

Macrophages are among the most abundant normal cells in the tumor immune microenvironment (86, 87). Macrophages that infiltrate the tumor microenvironment are often identified as tumor-associated macrophages (TAMs) (87). Macrophages alter their metabolic pathways, leading to their differentiation into inflammatory macrophages(M1) or regulatory macrophages (M2) subtypes in response to various cytokines (88). Given the important role of TAMs polarization in tumor development, it is important to study the effects of epigenetics on TAMs polarization.

**Figure 5:**
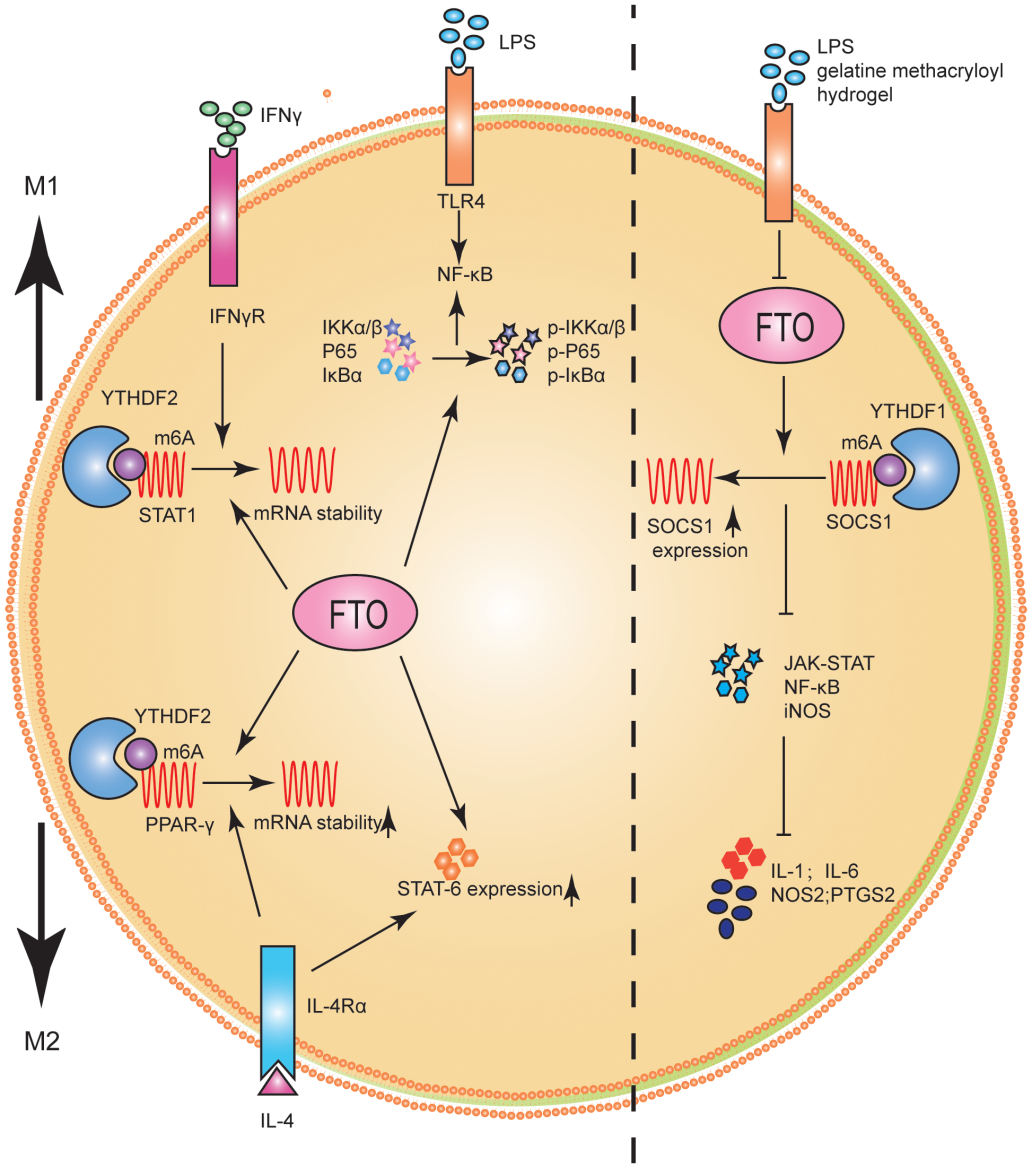
FTO promoted M1 and M2 macrophage activation. FTO promoted the polarization of M1 macrophages by improving STAT1 mRNA stability and enhancing the phosphorylation of IKKα/β, IκBα and P65. FTO promoted the polarization of M2 macrophages by enhancing the stability of PPAR-γ mRNA and increasing the expression of STAT6. FTO KO macrophages can reduce inflammatory responses by increasing m6A levels in Socs1 mRNA. YTHDF1 was identified as an m6A reader that recognizes Socs1 mRNA and enhances its stability, thus facilitating its translation.

Studies on the role and mechanism of FTO in macrophage polarization have shown that FTO significantly promoted M1 and M2 polarization (86). FTO inhibition decreased the phosphorylation levels of IKKα/β, IκBα, and P65, key genes of the NF-κB signaling pathway (86). It has been confirmed experimentally that STAT1 was an important molecule for the polarization of M1, and the levels of STAT6 and PPAR-γ represented the polarization degree of M2 (86, 89). Further studies showed that STAT1 expression level was significantly down-regulated in M1 macrophages after FTO inhibition, while PPAR-γ and STAT6 expression levels were inhibited in M2 macrophages. From the molecular mechanism, FTO inhibition promoted the decay of PPAR-γ and STAT1 mRNA. Furthermore, the expression and stability levels of STAT1 and PPAR-γ mRNA were significantly enhanced when m6A reader YTHDF2 was inhibited. The results suggested that FTO activated the NF-κB signaling pathway and promoted macrophage polarization by increasing the mRNA stability of PPAR-γ and STAT1 (86) (**Figure 5**).

Changes in macrophage activation and stiffness often represent significant pathological changes in tissues (90). For example, inflammatory tissue and tumors are often stiffer than healthy tissue (91, 92). Hu et al. (93) studied the potential role of FTO in macrophage activation and stiffness perception. FTO knockout (KO) macrophages reduced inflammatory responses by increasing m6A levels in Socs1 mRNA. YTHDF1 was identified as an m6A reader that recognized Socs1 mRNA and enhanced its stability, facilitating its translation (**Figure 5**).

According to recent studies on FTO and macrophages, FTO played an essential role in macrophage activation. Therefore, to further study how FTO affects the tumor immune microenvironment through macrophages, we need to co-culture tumor cells and macrophages based on FTO knockdown or overexpression and observe functional changes in macrophages.

## FTO as a therapeutic target

Given the critical role of FTO in tumor genesis and development, the development of small molecule inhibitors of FTO is a promising research direction. With the continuous understanding of FTO abnormal expression and its pathological mechanisms, as well as the clarification of the crystal structure of FTO (8, 24), developing small molecule compounds targeting FTO is possible (94). Several FTO inhibitors have been identified, and their therapeutic efficacy has been verified in various malignancies.

FB23, a novel MA-derived inhibitor, is nearly 140 times more active than MA at inhibiting FTO enzymatic activity (95). FB23-2 significantly inhibited proliferation and promoted differentiation and apoptosis of AML cells *in vitro*. Furthermore, FB23-2 drastically suppressed the progression of AML cells in xenotransplanted mice (95, 96). The function of FTO-04, a potent inhibitor of FTO, has also been demonstrated in glioma stem cells (GSC). Specifically, FTO-04 impaired the self-renewal of GSC-derived neural spheres but had no significant effect on healthy human neural stem cells (97).

The role of FTO inhibitors in regulating the tumor immune microenvironment is gaining attention. CS1 and CS2, two potent and selective FTO inhibitors, were found to have more satisfactory antitumor efficacy than FB32. Furthermore, CS1 and CS2 inhibited the self-renewal of leukemia-initiating cells and suppress immune infiltration by decreasing LILRB4 mRNA stability (18). Moreover, Dac51 was a recently discovered analog of FB23, that can weaken glycolytic metabolism and promote tumor tissue infiltration of CD8+T cells by inhibiting FTO, thus producing significant anti-tumor effects in melanoma (98). In summary, we believe that through continued efforts in the future, better quality FTO-targeted inhibitors will be discovered and applied clinically, bringing new hope for tumor immunotherapy.

## Discussion and future direction

Further exploration of the molecular mechanisms by which FTO regulates the tumor immune microenvironment, such as immune checkpoint expression, glucose metabolism and MSI, has valuable potential for clinical applications in guiding the selection of individualized treatment methods. Based on recent studies, small molecules or drugs targeting FTO may be effective in treating various malignancies. However, no direct FTO inhibitors have been approved for clinical use. Understanding how FTO affects tumor immunity should help clarify which patients with FTO-induced malignancies are most likely to respond to specific treatments, such as direct FTO inhibitors and FTO suppression combined with immunotherapy.

As the first discovered m6A demethylase, FTO has gained popularity in epigenetic research owing to its important roles in metabolism, tumors, and other physiological functions. To the best of our knowledge, the role and mechanism of FTO in tumor genesis and development have not yet been thoroughly studied. The important role of FTO in the tumor immune microenvironment still has a vast space to explore. Specifically, FTO inhibition reduced the expression of PD-1, CXCR4, and SOX10, which suggested that the combination of FTO inhibition with anti-PD-1 blockade may reduce the resistance to immunotherapy in melanoma (99). FTO regulated another important immune checkpoint, LILRB4, in leukemia cells (18). Bioinformatic analysis of FTO showed that the expression levels of other immune checkpoints, such as CTLA4, were significantly associated with m6A levels in multiple tumors (100, 101). Therefore, whether FTO can modulate other immune checkpoints in an m6A-dependent manner is an exciting research topic. MSI is a common gene mutation in malignant tumors and plays a vital role in many tumors (102). MSI of FTO can produce a mysterious polypeptide, FSP11, which promoted the infiltration of CD8+T cells, thereby affecting tumor progression (77). We already know that MSI is a pivotal predictor of immunotherapeutic regimen efficacy. Zhao et al. (103)extracted the MSI data from TCGA database. The results showed that the MSI significantly correlated with FTO expression in testicular germ cell tumors. Simultaneously, MSI was negatively associated with invasive breast carcinoma, thyroid carcinoma, head and neck squamous cell carcinoma, lung squamous cell carcinoma, stomach adenocarcinoma, prostate adenocarcinoma, cutaneous melanoma, and diffuse large B-cell lymphoma.

Of note, FTO affected tumor progression by regulating the efficiency of glucose metabolism (98, 104–106). For example, R-2HG, a specific FTO inhibitor, inhibited glycolysis by down-regulating the expression of two glycolytic enzymes, phosphofructokinase platelets and lactate dehydrogenase B. Furthermore, R-2HG exhibited strong antitumor activity based on the inhibition of glucose metabolism (104).

In most studies, FTO is overexpressed and plays a carcinogenic role, whereas in a few studies, FTO may also play a tumor suppressor role. Ruan et al. (107) studied the physiological role of FTO in the hypoxic environment of CRC and found that hypoxia induced FTO degradation via the ubiquitination-proteasome pathway. FTO inhibition increased m6A methylation of MTA1 mRNA, which was recognized by the m6A “reader” IGF2BP2, and maintained RNA stability/protein expression, thereby accelerating cancer metastasis and progression. Regarding tumor initiation, FTO has a protective effect on chemically induced HCC development. FTO may target cullin4A (Cul4A) mRNA and degrade Cul4A protein levels, thereby blocking cell proliferation (105).Huang et al. (106) found that FTO inhibited the expression of APOE through m6A modification mediated by IGF2BP2 and may inhibit the glycolytic metabolism of PTC by regulating the IL-6/JAK2/STAT3 signaling pathway, thus inhibiting tumor growth. Zhuang et al. (108) found that low FTO expression in human clear cell renal cell carcinoma (ccRCC) was associated with increased tumor severity and poor patient survival. FTO increased PGC-1α expression by reducing m6A levels in PGC-1 αmRNA transcripts, thereby restoring mitochondrial activity, inducing ROS production and oxidative stress, and inhibiting ccRCC growth. These conflicting results may be caused by individual differences in tumor patients, high heterogeneity of different cell lines of the same tumor, different experimental methods and equipments, and other potential biases.

Here, we summarize and discuss recent findings on the relevance of FTO in tumor immune microenvironment. Furthermore, we gain a deeper understanding of the molecular mechanisms by which FTO promoted GIC occurrence and development. Given the key role of FTO in GIC, targeting FTO as a therapeutic strategy is reasonable. Several studies have shown that FTO significantly affected the efficacy of tumor chemotherapy (12, 109, 110). Finally, we highlight targeting FTO strategies that may improve therapeutic outcomes for GIC, including combined immunotherapy.

## Conclusion

m6A is considered the most common epigenetic modification of RNA and plays an essential role in malignant cell proliferation, migration, invasion, and apoptosis. As the earliest demethylase discovered, FTO may initiate and maintain cancer through intrinsic carcinogenic signaling pathways and the immune microenvironment of tumor cells. In this review, we describe various mechanisms by which FTO regulates immune checkpoints and immune cells. Therefore, we believe that FTO can be used as biomarkers and adjuvant immunotherapy targets for GIC.

## Author contributions

XR conceptualized and designed the study. XR, XT, and TH drafted the work for intellectual content and context. ZH revised the first draft. YZ and YW gave the final approval and overall responsibility for the published work. All authors contributed to the article and approved the submitted version.
